# Can acupuncture increase microcirculation in peripheral artery disease and diabetic foot syndrome? – a pilot study

**DOI:** 10.3389/fmed.2024.1371056

**Published:** 2024-02-27

**Authors:** Jan Valentini, Martin Sigl, Cornelia Dunckel, Johannes Krisam, Klaus Amendt, Henry Johannes Greten

**Affiliations:** ^1^Institute of General Practice and Interprofessional Care, University Hospital Tuebingen, Tuebingen, Germany; ^2^First Department of Medicine, Division of Angiology, Faculty of Medicine of the University of Heidelberg, University Medical Center Mannheim, Mannheim, Germany; ^3^Practice for Traditional Chinese Medicine & Western Medicine, Oberschleissheim, Germany; ^4^Institute of Medical Biometry, Department Medical Biometry, University of Heidelberg, Heidelberg, Germany; ^5^Heidelberg School of Chinese Medicine, Heidelberg, Germany

**Keywords:** peripheral artery disease (PAD), diabetes mellitus, acupuncture, chronic wounds, microcirculation, diabetic foot syndrome (DFS)

## Abstract

**Background:**

Globally, diabetes mellitus (DM) and peripheral artery disease (PAD) have an increasing incidence and a high prevalence and are both associated with high morbidity and complication rates, e.g., as chronic non-healing peripheral ulcers. Impaired macro- and microcirculation and peripheral neuropathy lead to an increased risk of foot ulcers and infections. These complications are difficult to treat, have a high risk of becoming chronic and often lead to lower limb amputation. The aim of this planned study was to investigate the potential effects of acupuncture on improving microcirculation in patients with Diabetic Foot Syndrome (DFS) and PAD.

**Materials and methods:**

In 18 patients with chronic non-healing peripheral ulcers and diagnosed DM or PAD, data on 8 microcirculatory parameters were collected simultaneously on intact skin close to the wound margin. Microcirculation was assessed using an O2C device combining laser Doppler shift and white light spectroscopy (LEA Medizintechnik GmbH, Giessen, Germany). Unilateral and bilateral acupuncture was performed on the connecting line between acupuncture points Stomach 14 and Stomach 15.

**Results:**

After unilateral acupuncture (ipsilateral to the wound side), a statistically significant improvement in 7 out of 8 microcirculatory parameters was demonstrated compared to baseline measurements before acupuncture. After bilateral acupuncture, there was an additional improvement and statistical significance in all parameters in both DFS and PAD patients.

**Discussion:**

These results show an improvement in the microcirculation and peripheral blood flow at the edges of the wound. As impaired micro- and macrocirculation is considered to be a critical prognostic factor for the healing of a peripheral lesion, the intervention could have a positive impact on the healing of (chronic) peripheral wounds.

## 1 Introduction

Diabetes mellitus (DM) and peripheral artery disease (PAD) have an increasing incidence and a high prevalence worldwide. While around half a billion people currently suffer from diabetes, this figure is estimated to rise to more than three-quarters of a billion by 2045 (1). The situation is similar for PAD, the prevalence grew between 2000 and 2010 by 24% (2). In the meantime, far more than 200 million individuals are affected worldwide. The prevalence increases continuously with age. In high-income countries, it is estimated to be under 4% for men and women aged 40 to 44 years and over 20% for those over 80 years. The PAD prevalence is expected to continue to rise (3–5).

DM and PAD are both associated with high morbidity. Chronic non-healing peripheral ulcers, eventually combined with wound infections, are common in both patients with DM and patients with PAD. Diabetic foot syndrome (DFS) is one of the most clinically significant complications in patients with DM. Impaired blood flow and peripheral neuropathy increase the risk for foot ulcers and infections (6). People with diabetes mellitus have an estimated lifetime incidence of foot ulcers of up to 34% with frequent recurrences (7).

This is associated with a high economic burden. In the US, the treatment of diabetic foot syndrome is as expensive as that of carcinomas, with a comparable 5-year mortality rate (8). Recently, a large-scale study in Southeast Asia showed that coordinated care of patients with DFS from primary to tertiary care effectively reduces amputation rates (9). The data support the demand for a multi-disciplinary and interprofessional team approach in the management of DFS and lower extremity amputation prevention, as recommended by both the National Institute for Health and Care Excellence (NICE) and International Working Group on Diabetic Foot (IWGDF) guidelines (10, 11).

A common basic principle for the healing of a peripheral lesion is the restoration of impaired macro- and microcirculation, as this is considered to be a critical prognostic factor for wound healing (12–14). However, despite successful restoration of the macrocirculation, chronic wounds fail to heal in many cases. In a systematic review, wound healing was not achieved in 40% within 1 year in patients with DFS and PAD treated by endovascular or surgical therapy, and the major amputation rate was up to 10% (15). In addition, new treatment strategies are urgently needed for extremities at risk of amputation which cannot be revascularized for various reasons (16). In recent years, the crucial importance of microcirculation for limb preservation in both DFS and PAD has been emphasized. There is clear evidence that the microvascular function is altered first in the presence of risk factors associated with atherosclerosis (17). Even at the stage of intermittent claudication, microcirculation plays an important pathophysiological role (18). Patients with either diabetes or PAD demonstrate deteriorated cutaneous oxygen saturation at the plantar foot (19). Impaired microcirculation increases the risk of amputation in individuals with PAD by more than 20 times compared to those who neither have PAD nor microvascular disease (20). Therefore, interventions to improve cutaneous tissue oxygenation may be considered valuable therapy approaches to avoid complications and to promote the healing of chronic, non-healing peripheral ulcers.

One approach that might meet these criteria is acupuncture. It is a therapy from Traditional Chinese Medicine (TCM) and can be understood as reflex zone therapy, where certain areas of the body are stimulated with acupuncture needles (21, 22). Although the mechanisms of action of acupuncture have not yet been fully scientifically clarified, there are numerous publications that prove the effectiveness and efficacy of acupuncture (23–28). According to current studies, acupuncture can be used to increase peripheral microcirculation and skin blood perfusion (29–33). This effect may be useful in the therapy of DM and PAD patients with chronic ulcers. The aim of the study therefore was to investigate potential effects of acupuncture on microcirculation close to the wound margin in patients with DM or PAD.

## 2 Methods

### 2.1 Study design

We conducted a prospective, non-controlled pilot study to assess changes in microcirculation before and after intervention in patients with DM and PAD following unilateral or ipsilateral acupuncture.

The study was reviewed and approved by the Medical Ethics Committee II of the Mannheim Medical Faculty under the numbers 2013–546 N-MA and 2013–547 N-MA for the study in patients with PAD and DFS, respectively.

### 2.2 Study population

Inpatients from the Department of Angiology and Diabetes-related Diseases at the Diakonissenkrankenhaus Mannheim, Germany, were selected according to the following inclusion and exclusion criteria.

#### 2.2.1 Inclusion criteria

Patients with peripheral lesions of the lower limbs and diagnosed PAD Fontaine stages III-IV (34, 35) or DFS, Wagner-Armstrong classification stages 1–4 A-D (36) were included in the study. All study participants provided written informed consent in German language.

#### 2.2.2 Exclusion criteria

Exclusion criteria were angiological intervention (e.g., percutaneous transluminal angioplasty or bypass surgery) within the previous 2 months, acute ischemia, vasoactive therapy within the previous 48 h, body temperature > 37.8°C or systemic signs of infection. Other exclusion criteria were skin disease in the area of the acupuncture point, oral anticoagulation therapy and women of childbearing age.

### 2.3 Intervention

The intervention is reported according to the STRICTA guidelines (37). Acupuncture was provided by two physicians with an additional specialization in acupuncture from the Medical Chamber Baden-Württemberg, Germany, or an equivalent of at least 200 teaching hours. Each acupuncturist had more than 10 years’ experience in acupuncture and TCM. The acupuncture treatment consisted of a single acupuncture session of approximately 5 minutes’ duration. The acupuncture was performed on the connecting line between the acupuncture points Stomach 14 (ST 14, 库房, kù fáng) and Stomach 15 (ST 15, 屋 翳, wū yì) (22). These points are located at the front of the chest in the first and second intercostal space respectively, on the mamillary line. The selected points were chosen based on principles of Chinese medical theory and supported by available evidence, as follows: (i) According to Chinese medical theory, points are selected based on their location along the same meridian (38). Acupuncture points on the Stomach meridian were chosen to target the back of the foot, where many chronic wounds in PAD and DFS are located. The Stomach meridian starts at the lateral side of the nose, runs through the chest and abdomen, extends to the front of the upper and lower leg, and ends at the back of the foot on the second toe (22). (ii) Additionally, a fundamental principle of acupuncture emphasizes the combination of local and distal points. Due to a relative contraindication for needling close to open wounds, only distal points were chosen for needling (22). (iii) The decision to use the region between these two points on the chest was further supported by clinical experience and observations within the author group, as understood in terms of internal evidence. Acupuncture was applied either unilaterally (ipsilateral to the wound side) or bilaterally (first ipsi- then contralateral) to the other half of the participants. A so-called sparrow-picking or blood-letting technique was used with standardized needles (Becton Dickinson B-D Micro Fine ™ + Demi insulin syringes 0.3 × 5 mm, 31 G) (32, 39, 40). The needles were inserted to their full length of 5 mm, no needle retention was used in this technique. No specific response (e.g., de qi) was sought. No other interventions (e.g., moxibustion, cupping, lifestyle advice) were given to the participants.

### 2.4 Micro-lightguide spectrophotometer (O2C)

Microcirculation was assessed using an Micro-lightguide Spectrophotometer (O2C device), which combines a laser Doppler shift and white light spectroscopy system (LEA Medizintechnik GmbH, Giessen, Germany) (41). A flat probe placed horizontally on the tissue collects data simultaneously from the tissue as a multi-channel system. Data collection was performed on the intact skin at the distance of 1 cm from the wound edge of the damaged tissue and covered a time interval from 3 min before acupuncture (= baseline measurement) until 10 min after acupuncture. Due to potential movement artifacts, data recorded 30 s each before and after the acupuncture treatment were excluded from data analysis. Microcirculation was assessed using the following microperfusion parameters: oxygen saturation of hemoglobin (SO2), relative amount of hemoglobin (rHb), relative blood flow (Flow) and blood flow velocity (Velo). SO2 was expressed as a percentage, rHb, Flow and Velo in arbitrary units (AU). These four parameters were measured at 3 mm (S) and 8 mm (D) depth, resulting in a total of 8 parameters (e.g., Velo S (superficial 3 mm) and Velo D (deep 8 mm)). In cases of diabetic microangiopathy, the O2C device has been shown to provide reliable data and valid non-invasive measurements of tissue oxygenation and microvascular blood flow (42, 43).

### 2.5 Statistical analysis

Linear mixed models were used to assess the influence of acupuncture on the eight microcirculatory parameters. These models included each measured parameter as a dependent variable, acupuncture (baseline/unilateral acupuncture/ bilateral acupuncture) and disease (PAD/DFS) as fixed factors, and patient as a random factor. The inclusion of patient as a random factor ensures that the clustered data structure is considered in the statistical model (measurements clustered within patients). An additional linear mixed model explored the difference in the effects of acupuncture between PAD and DFS patients by including an interaction term between acupuncture and disease. Restricted maximum likelihood was used to fit all models. Effect estimates were calculated along with *p*-values and 95% confidence intervals of the profile likelihood type. Due to the exploratory nature of the study, no adjustment for multiple testing was made. *P*-values less than 0.05 were considered statistically significant. R version 3.4.2^1^ with the packages ‘lme4’ and ‘lmerTest’ was used for statistical analysis.

## 3 Results

### 3.1 Patient characteristics: sociodemographic and clinical characteristics

A total of *n* = 18 patients were included in the pilot study, thereof *n* = 9 with DFS and *n* = 9 with PAD. Two thirds of the participants were male (*n* = 12) and one third female (*n* = 6). Age of the participants ranged from 55 to 92 years, with a mean of 77 years (SD 9.5). Additional clinical characteristics of the study population are shown in Table 1.

**Table 1:** Sociodemographic data: description of the study population.

Characteristics		*n* = 18	[%]
Disease	PAD	9	50
	DFS	9	50
Gender	Male	12	66.7
	Female	6	33.3
Acupuncture	Ipsilateral	8	44.4
	Ipsi- and contralateral	10	56.6
Age [mean (SD)]		76.94 (9.5), min 55, max 92	

SD, Standard Deviation.

### 3.2 Unilateral and bilateral acupuncture in pre-post comparison in patients with DFS and PAD

After unilateral acupuncture (ipsilateral to the wound side), a statistically significant improvement in seven out of eight microcirculatory parameters (superficial and deep Flow, Velo and rHb as well as in deep S02) could be demonstrated in both patients with PAD and DFS with chronic non-healing peripheral ulcers compared to baseline measurements before acupuncture. With unilateral acupuncture alone, superficial SO2 measurements did not show a statistically significant improvement. However, bilateral acupuncture (first ipsi-, then contralateral) led to statistically significant improvements in all eight microcirculatory parameters s compared to baseline measurements. When comparing unilateral versus bilateral acupuncture in patients with both DFS and PAD, an additional improvement in all parameters was observed after bilateral acupuncture. Further details are shown in Tables 2, 3.

**Table 2:** Difference between baseline measurements (Base), unilateral acupuncture (Acu 1) and bilateral acupuncture (Acu 2).

Parameter			Estimate	Std. Error	value of p	95% Confidence Interval	
						Lower bound	Upper bound
Flow	Flow S	Acu 1 vs. Base	12.08	1.12	**< 0.001**	9.89	14.27
		Acu 2 vs. Base	18.16	1.36	**< 0.001**	15.49	20.83
	Flow D	Acu 1 vs. Base	9.33	1.10	**< 0.001**	7.18	11.48
		Acu 2 vs. Base	20.92	1.34	**< 0.001**	18.29	23.54
SO2	SO2 S	Acu 1 vs. Base	0.34	0.26	0.186*	−0.16	0.85
		Acu 2 vs. Base	1.67	0.32	**< 0.001**	1.05	2.29
	SO2 D	Acu 1 vs. Base	1.57	0.23	**< 0.001**	1.12	2.03
		Acu 2 vs. Base.	3.65	0.28	**< 0.001**	3.10	4.21
Velo	Velo S	Acu 1 vs. Base	1.11	0.15	**< 0.001**	0.82	1.40
		Acu 2 vs. Base	1.71	0.18	**< 0.001**	1.36	2.07
	Velo D	Acu 1 vs. Base	1.14	0.16	**< 0.001**	0.83	1.45
		Acu 2 vs. Base	2.74	0.19	**< 0.001**	2.36	3.12
rHb	rHb S	Acu 1 vs. Base	0.44	0.22	**0.0456**	0.01	0.88
		Acu 2 vs. Base	1.90	0.27	**< 0.001**	1.37	2.42
	rHb D	Acu 1 vs. Base	3.02	0.37	**< 0.001**	2.29	3.74
		Acu 2 vs. Base	5.78	0.45	**< 0.001**	4.89	6.66

Acu 1, unilateral acupuncture; Acu 2, bilateral acupuncture; Base, baseline readings before acupuncture; *p* value: <0.05 statistical significance showed in bold.

* = not statistically significant.

**Table 3:** Difference between unilateral acupuncture (Acu 1) and bilateral acupuncture (Acu 2).

Parameter			Estimate	Std. Error	value of p	95% Confidence Interval	
						Lower bound	Upper bound
Flow	Flow S	Acu 2 vs. Acu 1	6.08	1.36	**< 0.001**	3.41	8.75
	Flow D	Acu 2 vs. Acu 1	11.59	1.34	**< 0.001**	8.96	14.21
SO2	SO2 S	Acu 2 vs. Acu 1	1.32	0.32	**< 0.001**	0.70	1.94
	SO2 D	Acu 2 vs. Acu 1	2.08	0.28	**< 0.001**	1.53	2.63
Velo	Velo S	Acu 2 vs. Acu 1	0.61	0.18	**< 0.001**	0.25	0.96
	Velo D	Acu 2 vs. Acu 1	1.59	0.19	**< 0.001**	1.22	1.97
rHb	rHb S	Acu 2 vs. Acu 1	1.45	0.27	**< 0.001**	0.93	1.98
	rHb D	Acu 2 vs. Acu 1	2.76	0.45	**< 0.001**	1.87	3.64

Acu 1, unilateral acupuncture, Acu 2, bilateral acupuncture, *p* value: <0.05 statistical significance showed in bold.

### 3.3 Acupuncture in patients with DFS versus PAD

**Figure 1:**
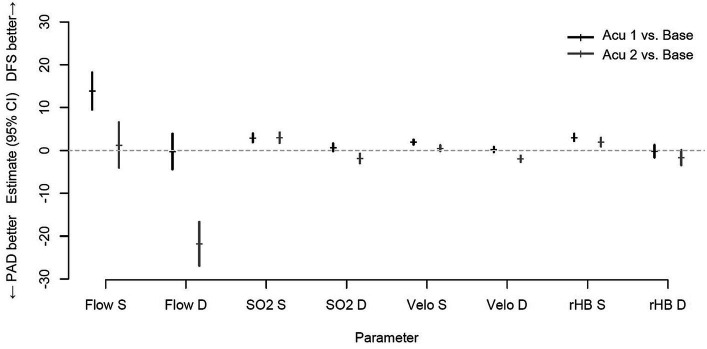
Mean outcome differences with 95% confidence intervals in acupuncture intervention between patients with PAD and DFS in unilateral acupuncture (Acu 1) and bilateral acupuncture (Acu 2).

Table 4 shows the different results of the acupuncture intervention in patients with PAD compared to patients with DFS for unilateral and bilateral acupuncture compared to baseline measurements before acupuncture. For all superficial measurements (3 mm depth), better results could be shown for single and bilateral acupuncture in patients with DFS compared to patients with PAD (values marked with ∞ in Table 4). For the deep measurements (8 mm depth), an inverse trend could be found. After unilateral acupuncture, SO2 and Velo measurements were better in patients with DFS compared to PAD. On the contrary, Flow and rHb measurements were better in PAD compared to DFS (values marked with ∆ in Table 4). Measurements of blood flow showed the most distinct differences between patients with DFS and PAD. In addition, with bilateral acupuncture, all deep measurements showed better results in PAD than in DFS. Although there were some trends, not every difference shown between patients with DFS and PAD reached statistical significance. Figure 1 shows the mean values for the differences in treatment effect along with 95% confidence intervals in the acupuncture intervention between patients with PAD and DFS in unilateral acupuncture and bilateral acupuncture when compared to baseline measurements before acupuncture.

**Table 4:** Difference in acupuncture intervention for patients with DFS versus PAD in unilateral acupuncture (Acu 1) and bilateral acupuncture (Acu 2) when compared to baseline measurements before acupuncture.

Parameter				Estimate	Std. Error	*p*-value	95% Confidence Interval	
							Lower bound	Upper bound
Flow	Flow S	Acu 1	DFS vs. PAD	13.86^∞^	2.20	**< 0.001**	9.55	18.17
		Acu 2		1.26^∞^	2.71	0.642*	−4.04	6.56
	Flow D	Acu 1	DFS vs. PAD	−0.25^∆^	2.13	0.907*	−4.42	3.92
		Acu 2		−21.80^∆^	2.62	**< 0.001**	−26.93	−16.69
SO2	SO2 S	Acu 1	DFS vs. PAD	2.94^∞^	0.51	**< 0.001**	1.94	3.94
		Acu 2		2.96^∞^	0.63	**< 0.001**	1.73	4.19
	SO2 D	Acu 1	DFS vs. PAD	0.712^∞^	0.46	0.122*	−0.19	1.61
		Acu 2		−1.87^∆^	0.57	**0.001**	−2.98	−0.76
Velo	Velo S	Acu 1	DFS vs. PAD	1.95^∞^	0.29	**< 0.001**	1.38	2.52
		Acu 2		0.53^∞^	0.36	0.139*	−0.17	1.23
	Velo D	Acu 1	DFS vs. PAD	0.21^∞^	0.31	0.499*	−0.40	0.83
		Acu 2		−1.97^∆^	0.39	**< 0.001**	−2.73	−1.22
rHb	rHb S	Acu 1	DFS vs. PAD	3.03^∞^	0.43	**< 0.001**	2.18	3.88
		Acu 2		1.94^∞^	0.53	**< 0.001**	0.89	2.98
	rHb D	Acu 1	DFS vs. PAD	−0.20^∆^	0.74	0.784*	−1.66	1.25
		Acu 2		−1.69^∆^	0.91	0.064*	−3.47	0.10

Acu 1, unilateral acupuncture, Acu 2, bilateral acupuncture.

^∞^ values = DFS better than PAD.

^∆^ values = PAD better than DFS.

*p* value: <0.05 statistical significance showed in bold.

*not statistically significant.

## 4 Discussion

After unilateral acupuncture (ipsilateral to the wound side), a statistically significant improvement in seven out of eight microcirculatory parameters (SO2, rHb, Flow and Velo) was demonstrated at 3 mm and 8 mm depths compared to baseline measurements before acupuncture. These results correspond to an increase in microcirculation around the wound margin. Impaired micro- and macrocirculation is considered a critical prognostic factor for the healing of a peripheral lesion (12–14). Beside arterial revascularization as main therapeutic option, there is limited evidence for non-revascularization interventions including, e.g., Prostanoids (16). Although recommended to accelerate ulcer healing, pain reduction and amputation prevention in PAD (44, 45), Alprostadil (prostaglandin E1) failed to show superiority over placebo in a placebo-controlled randomized multicenter trial (46). Therefore, the results of this study must be interpreted with great caution when considering their clinical implications in wound healing. Nevertheless, acupuncture may have a positive influence on wound healing in (chronic) peripheral wounds. As numerous studies have explored acupuncture’s impact on microcirculation, including capillary blood flow, indicating potential improvements under specific conditions (47–49). However, the precise mechanisms underlying acupuncture’s effects on microcirculation remain under investigation. Some research proposes various mechanisms, including the release of neurotransmitters (e.g., nitric oxide), activation of mast cells and regulation of the autonomic nervous system among others (50–55). When comparing the results of the acupuncture intervention between patients with DFS and PAD, better results could be shown for superficial measurements in patients with DFS. However, for deep measurements, a more pronounced improvement was seen in patients with PAD compared to DFS. Although these differences are only a trend, as they did not reach statistical significance for every microcirculatory parameter measured, the reason for these differences is not clear. Our findings in patients with DFS are consistent with those recently published by Sang et al., who showed that diabetic patients with arteriosclerotic wounds of the lower extremity had a significant reduction in lesion size after 8 weeks of treatment with electroacupuncture (56). An exact explanation of the precise acupuncture mechanisms of our observed effects can only be hypothesized. We would suggest that the observed results could be mainly due to regulatory effects of the autonomic nervous system, which is known to regulate, e.g., perfusion and vasodilation (57). On the other hand we would rather rule out that the effects are mainly due to effects on segmental innervation, as the needle insertion point was in the at the cervicothoracic region, whereas the observed effects on microcirculation were in the lumbar or pelvic innervation region. Further studies should address these issues.

### 4.1 Strengths and limitations

Owing to the exploratory design of this pilot study, there are several limitations that need to be considered when interpreting the results. Due to the small sample size, this study did not include a control group (e.g., sham acupuncture or a control group). This also led to a lack of randomization and blinding of patients. However, as the data recorded before the acupuncture intervention were considered as the baseline measurement, a comparison of the microcirculatory parameters before and after the acupuncture intervention could be implemented in the data analysis. As there was no follow-up in this study, only an immediate acupuncture effect could be demonstrated. As the study used a specific needling technique, it’s important to note that the results may not necessarily be applicable to other types of needling or the use of different stimulation methods. Therefore, it is not possible to say how long the effects of acupuncture may last. Whether the measured statistically significant improvement in microcirculatory parameters leads to a clinically significant improvement, resulting in faster healing of chronic peripheral wounds, remains uncertain. Further studies should include a larger sample size, a control group, different needling techniques and ideally, assessments of clinical parameters of wound healing (e.g., wound size with length and width) over a longer period of time, due to the chronicity of lower limb ulcers.

The strength of this study is the investigation of a pragmatic acupuncture approach with a comparatively feasible acupuncture intervention for a worldwide relevant problem of non-healing chronic peripheral wounds in patients with DFS or PAD. As no prior diagnosis according to TCM criteria is required before the acupuncture intervention, it may be a quick and effective method that could potentially be easily implemented and performed during routine medical consultations for both inpatients and outpatients. As no adverse events were reported during the trial, this acupuncture intervention can be considered safe, despite the small sample size. Based on the extensive data available, the overall risk of complications from acupuncture is considered to be very low. No adverse events (e.g., pneumothorax) have been reported in the literature for the acupuncture points used, Stomach 14 and Stomach 15 (58, 59).

These results show an improvement in the microcirculation and peripheral blood flow at the edges of the wound. As impaired micro- and macrocirculation is considered to be the most important prognostic factor for the healing of a peripheral lesion, the intervention may have a positive impact on the healing of (chronic) peripheral wounds.

## Data availability statement

The raw data supporting the conclusions of this article will be made available by the author on reasonable request in anonymised form in accordance with the institutional regulations and the General Data Protection Regulation.

## Ethics statement

The studies involving humans were approved by Medical Faculty Mannheim, Medical Ethics Commission II (Nr. 2013-546N-MA and 2013-547N-MA). The studies were conducted in accordance with the local legislation and institutional requirements. The participants provided their written informed consent to participate in this study.

## Author contributions

JV: Conceptualization, Data curation, Investigation, Writing – original draft. MS: Investigation, Writing – review & editing. CD: Investigation, Writing – review & editing. JK: Data curation, Formal analysis, Methodology, Validation, Visualization, Writing – review & editing. KA: Conceptualization, Resources, Supervision, Validation, Writing – review & editing. HG: Conceptualization, Resources, Supervision, Writing – review & editing.

## Footnotes

^1^http://r-project.org
